# Lower Extremity Injuries in Elite Snowsport Athletes: A Retrospective Survey

**DOI:** 10.3390/jcm15020695

**Published:** 2026-01-15

**Authors:** Buket Sevindik Aktas, Esedullah Akaras, E. Whitney G. Moore, Ersagun Kepir, Anthony Kulas, Gokhan Yagiz

**Affiliations:** 1 Faculty of Sport Sciences, Erzurum Technical University, Erzurum 25050, Türkiye; 2 Department of Physiotherapy and Rehabilitation, Faculty of Health Sciences, Erzurum Technical University, Erzurum 25050, Türkiye; esedullah.akaras@erzurum.edu.tr; 3 Department of Kinesiology, College of Health and Human Performance, East Carolina University, Greenville, NC 27858, USA; mooreer22@ecu.edu (E.W.G.M.); kulasa@ecu.edu (A.K.); 4 Department of Physiotherapy and Rehabilitation, Faculty of Health Sciences, Çukurova University, Adana 01330, Türkiye; ersagunkepir@gmail.com; 5 Department of Physiotherapy and Rehabilitation, Faculty of Health Sciences, Amasya University, Amasya 05100, Türkiye; gokhan.yagiz@amasya.edu.tr; 6 Department of Physical Therapy, Faculty of Health Sciences, Tokyo Metropolitan University, Tokyo 92-0397, Japan

**Keywords:** lower extremity injuries, snowsports, injury incidence, injury burden, skiing

## Abstract

**Background/Objectives**: Lower extremity injuries represent a major health concern in elite snowsport disciplines, where high mechanical loads, complex movement patterns, and demanding environmental conditions substantially increase injury risk. Understanding injury incidence and burden in this population is essential for developing sport- and sex-specific prevention strategies. This retrospective study determined lower extremity injury incidence and burden among elite snowsport athletes. **Methods**: Ninety-nine Turkish National Snowsport Teams Training Camp athletes (34 females; 65 males) consented to a review of their medical records for injury incidence. Overall, sex- and sport-specific injury incidence (number/10,000 h) and burden (weeks missing/10,000 h) were calculated. **Results**: Overall, medial tibial stress syndrome (MTSS) was the highest burden (9.5 ± 38.7), and ankle sprain (1.7 ± 0.4) was the highest-incident injury. However, injury incidence and burden patterns differed by sex and sport. Notably, medial tibial stress syndrome (MTSS) showed comparable incidence in female and male athletes but resulted in a substantial injury burden in both sexes, reflecting prolonged time-loss from training and competition and indicating a meaningful negative impact on athletic performance. Specifically, the highest-burden injury for women was anterior cruciate ligament (ACL) rupture (16.2 ± 64.5), and for men the most common injury was MTSS (9.7 ± 40.7). For cross-country skiers, MTSS had the highest burden and incidence. For all other sports, and across sexes, ankle sprain was the highest incidence injury—women (1.3 ± 3.0), men (2.0 ± 4.5), biathletes (2.3 ± 5.7), Alpine skiers (2.8 ± 4.5), ski jumpers (1.6 ± 3.1), and snowboarders (3.2 ± 4.7)—plus the highest-burden injury for biathletes (6.9 ± 14.3) and ski jumpers (6.0 ± 14.0). The highest burden injury for Alpine skiers was ACL damage (34.3 ± 87.2), and for snowboarders it was knee collateral ligament injury (27.8 ± 78.6). Moreover, patellar tendinitis, hamstring strains, calf strains, Achilles ruptures, anterior tibial pain, meniscus tears, and hip injuries were frequently observed in injury patterns. **Conclusions**: Ankle sprains were the most frequent lower extremity injury in elite snowsport athletes, whereas medial tibial stress syndrome (MTSS) and anterior cruciate ligament (ACL) injuries accounted for the greatest injury burden. Injury incidence and burden differed by sex and snowsport discipline.

## 1. Introduction

Identifying the prevalence and occurrence of injuries is essential, and the increasingly long-lasting careers of athletes may increase the risk of injury prevention strategies. Lower extremity injuries are the most common and severe injuries in snowsports [1], such as in Alpine skiers [1,2], freestyle skiers [1], snowboarders [1], and cross-country skiers [3]. According to the International Ski Federation (FIS) Injury Surveillance System report (between 2006 and 2019) [4], among all injuries, lower extremity injuries constituted 57.8–59.7% in Alpine skiing (World and European Cup injuries, subsequently), 49.3% in freestyle skiing, 39.8% in snowboarding, and 69.7% in ski jumping. Similarly, a recent systematic review and meta-analysis [5] detected that 44% of all injuries were to the lower extremities during the Winter Olympics from 1995 through 2021. Given this information, lower extremity injuries demand a closer investigation among snowsport athletes.

Lower extremity injuries in snowsport athletes commonly include muscle and ligament tears, as well as bone fractures [1]. While these injuries are often classified according to the affected anatomical structures, they can also be broadly categorized based on their underlying mechanisms, namely acute traumatic injuries and overuse-related injuries. Overuse injuries, characterized by their occurrence without a specific, identifiable event, can pose a significant challenge in numerous sporting activities [6], and can initially appear as minor traumas (e.g., microtraumas), which can have cumulative impacts for developing overuse injuries [7,8,9]. In the long term, overuse injuries negatively affect athletes’ performance, sometimes leading to the end of their careers [9,10,11].

In contrast to acute and traumatic injuries, overuse injuries have garnered lesser attention in the literature on sports injury prevention [6,12]. The underlying reason for the lack of knowledge regarding overuse injuries is difficulties in recording them due to their characteristics [13]. Early symptoms of overuse injuries (e.g., functional limitations and pain) generally occur gradually, sometimes provisionally, and thus, athletes may keep their training routine in the early stages or adapt their form of exercise to avoid inconvenient activities [6]. Therefore, injury surveys often overlook minor injury characteristics, such as pain and functional limitations, which may serve as early indicators of more serious overuse conditions. Addressing these subtle signs could improve early prevention and monitoring strategies in elite skiing populations

Overuse injuries require particular attention in preventive efforts among skiers, as these injuries often develop gradually due to repetitive mechanical loading and may remain unrecognized in their early stages, thereby increasing the risk of performance impairment and long-term participation limitations if not adequately addressed [14]. Most existing injury surveys have primarily focused on major injuries, such as anterior cruciate ligament (ACL) ruptures, muscle strains, and ligament sprains. However, this study aimed to focus on both major and minor lower extremity injuries, including medial tibial stress syndrome (MTSS or shin splints), patellofemoral pain syndrome (PFPS), and patellar tendinitis (jumpers’ knee) to detect early signs of future injuries which may affect snowsport athletes’ sports performance and career longevity. In this way, revealing injury patterns may help broaden and optimize injury prevention strategies in snowsports [15]. Although this study provides a comprehensive overview of lower extremity injuries, particular attention is given to overuse injuries due to their cumulative burden and implications for long-term athlete health. However, the existing literature remains limited in providing comprehensive epidemiological data that jointly report injury incidence and injury burden across multiple snowsport disciplines, particularly with respect to overuse injuries and sex-specific patterns. The present study addresses this gap by offering a multi-discipline, sex-specific epidemiological analysis of lower extremity injuries in elite snowsport athletes.

## 2. Methodology

### 2.1. Study Design

This study employed a retrospective injury surveillance design and received ethical approval from the Atatürk University Ethics Commission (Number: 2022-9 E-70400699-050.02.04-2200285982). With permission from the athletes, medical records for their whole athletic career were examined, related to participation in the international winter sports games held in the Olympic Athlete Preparation Center of the Republic of Türkiye. Additionally, face-to-face interviews with the athletes were conducted in Erzurum to approve the sharing of medical records between 1 October 2022 and 1 May 2023. An injury was defined as any musculoskeletal condition that resulted in either complete cessation of training/competition or a medically advised reduction in training volume or intensity [16]. This study was conducted in accordance with the ethical principles of the Declaration of Helsinki, and written informed consent was obtained from all participants. Additionally, our data availability statement is clear.

### 2.2. Ethical Considerations

Ethical approval was granted before data collection, ensuring confidentiality and data protection. All injury data were anonymized prior to analysis, and only aggregate findings are presented.

### 2.3. Participants

In the present study, athletes were classified as elite snowsport athletes based on their active registration with the Turkish National Snowsport Teams, regular participation in national and international competitions, and engagement in structured, high-volume training programs conducted within the national Olympic preparation system. Ninety-nine elite level snowsports athletes (thirty-four females (34.3%) and sixty-five males (65.7%)) participated in the study. They represented five snow sports: forty-one cross-country skiers (41.4%), twenty-four biathlon (cross-country + rifle shooting) skiers (24.2%), eighteen Alpine skiers (18.2%), eight ski jumpers (8.1%), and eight snowboarders (8.1%). The inclusion criteria for participation in this study were as follows: (i) active membership in the Turkish National Snowsport Teams and recruitment from the national team training camp held at the Turkish National Olympic Preparation Center prior to national selection competitions; (ii) participation in official national and/or international snowsport competitions and eligibility for reselection to the national team; (iii) engagement in regular, systematic, and structured training programs within the Olympic Athlete Preparation Center; (iv) active competitive status at the time of data collection; (v) availability of complete and verifiable medical records covering the athlete’s competitive career.

### 2.4. Data Collection

After the relevant athletes filled out the voluntary consent form, their demographic information was recorded. The athletes’ total sporting years, national qualification times, and total training times were recorded. Medical records of the athletes were retrospectively examined, and athletes were asked to confirm the injuries and their details via face-to-face interviews about their injury history. Injury history, severity, injury-caused time loss, training exposure, injury incidence, and prevalence were recorded. In addition, the athletes’ age at first injury was also recorded. Injury incidences and burdens were calculated via the following formulas [17]. To estimate total exposure hours, documented annual training and competition schedules obtained from the Turkish National Snowsport Federation were used as the primary source, supported by face-to-face athlete interviews to verify participation continuity. Injury records and exposure estimates were not solely based on athlete recall. All available injury diagnoses, time-loss durations, and training exposure data were cross-validated using official medical and training records obtained from the Turkish Ski Federation. Federation records were prioritized in cases of discrepancies, and athlete interviews were used only to confirm participation continuity and return-to-sport dates. Exposure data were excluded only when information could not be reliably verified from either source. Each athlete’s total career exposure was calculated based on average weekly training hours and number of active years under national team registration. Discrepancies between athlete reports and official training logs were checked, and inconsistent data were excluded. Time-loss information used for calculating injury burden was primarily derived from official medical records, and athlete confirmation was sought only to validate return-to-sport dates. This methodological approach was adopted to minimize recall bias and to ensure reliability within the constraints of a retrospective design.


$$Injury\;incidence=Number\;of\;injuries\;per\;athlete\;per\;10,000\;h=\left(\frac{\Sigma injuries}{\Sigma exposure\;hours}\right)*10,000)$$

$$Injury\;burden=Number\;of\;layoff\;weeks\;per\;athlete\;per\;10,000\;h=\left(\frac{\Sigma lay-offweeks}{\Sigma exposure\;hours}\right)*10,000)$$

Total exposure hours of the athletes were calculated as their total elite athletic careers.

### 2.5. Statistical Analysis

Statistical analyses were conducted using SPSS software (IBM SPSS Statistics, version 29). Mean, standard deviation (SD), and 95% confidence interval (CI) values were calculated for each injury for the total sample, each sex, and snowsport type. We calculated 95% CIs using Poisson-distribution-based confidence intervals, following standard sports injury epidemiology methods. Although injury incidence is often expressed per 1000 h, we reported data per 10,000 exposure hours to allow comparability with similar retrospective and multi-discipline studies [6,18].

## 3. Results

The mean and SD for the participating athletes’ age, sex, body mass, height, body mass index (BMI), total years of athletic performance, age at first injury, total duration of national team membership in years, and total training/competition hours are shown in Table 1.

Table 2 presents, for each injury type, the total injury incidence per 10,000 h, total injury burden (number of time-off weeks per 10,000 h), competition injury incidence per 10,000 h, and the training injury incidence per 10,000 h. The compared study reported incidence per 1000 h, whereas our data are presented per 10,000 h to enable comparison across multiple disciplines and longer exposure periods. Overall, injury incidence during competition hours was low, and the incidence of injuries during competition was lower compared with that observed during training. Table 3 presents, by sport, the top five injuries by incidence plus by burden.

When injury incidence patterns were examined for the whole cohort, ankle sprains were identified as the most frequent injury, followed by medial tibial stress syndrome (MTSS), tibialis anterior pain, hip injuries, and Achilles tendon ruptures. Other injuries, including quadriceps femoris strains, patellar tendinitis, knee collateral ligament injuries, hamstring strains, meniscus tears, anterior cruciate ligament (ACL) ruptures, patellofemoral pain syndrome, and calf strains, occurred less frequently. In contrast to injury incidence, the distribution of injury burden showed a different pattern. MTSS accounted for the greatest overall injury burden, followed by ACL ruptures and ankle sprains, whereas the remaining injuries were associated with comparatively shorter time-loss durations (Table 2 and Table 3).

Among women athletes, ankle sprains were the most frequently occurring injury, while ACL ruptures were associated with the highest injury burden. MTSS represented the second most common injury and also contributed substantially to injury burden, followed by hamstring strains, calf strains, and Achilles tendon ruptures. In male athletes, ankle sprains were likewise the most common injury; however, MTSS resulted in the greatest time loss. Other injuries, including ankle sprains, knee collateral ligament injuries, patellar tendinitis, and tibialis anterior pain, contributed to injury burden to a lesser extent (Table 2 and Table 3).

Injury patterns also differed across snowsport disciplines. In cross-country skiers, MTSS was the most prominent injury in terms of both incidence and burden, followed by tibialis anterior pain, hip injuries, quadriceps femoris strains, and Achilles tendon ruptures. Among biathletes, ankle sprains were the most frequent injury and also accounted for the greatest injury burden, whereas hip injuries, patellar tendinitis, knee collateral ligament injuries, and MTSS contributed less substantially. Alpine skiers demonstrated a distinct profile, with ankle sprains occurring most frequently, while ACL ruptures resulted in the highest injury burden, followed by MTSS and patellar tendinitis. In ski jumpers, ankle sprains were the most common injury and also represented the leading contributor to injury burden, followed by patellar tendinitis and MTSS. Among snowboarders, ankle sprains were the most frequent injury; however, knee collateral ligament injuries accounted for the greatest injury burden, followed by ACL ruptures and ankle sprains (Table 2 and Table 3).

## 4. Discussion

This study focused on retrospectively revealing lower extremity injury incidences and the burden of the injuries among elite snowsport athletes of the Turkish National Snowsport Teams. The highest-incident injury was ankle sprain, and this situation did not change based on sex. In contrast, medial tibial stress syndrome (MTSS) emerged as the injury associated with the greatest overall injury burden, as reflected by prolonged time-loss from training and competition, and was followed in magnitude by anterior cruciate ligament (ACL) ruptures and ankle sprains. However, when considering sex and sport disciplines, the patterns of injury burden changed. Specifically, among women athletes ACL ruptures led to the highest burden. On the other hand, among male athletes, MTSS had the highest time-off weeks. It is worth noting that as a chronic injury, MTSS may have been missed in injury studies including only acute injuries. Our findings align with international injury surveillance reports from the International Ski Federation (FIS) and the International Olympic Committee (IOC), identifying lower extremity injuries as the most prevalent in elite snowsports. Differences in injury incidence and burden across disciplines likely reflect sport-specific biomechanical demands, with MTSS predominating in cross-country skiers, ACL injuries in Alpine skiers, and knee collateral ligament injuries in snowboarders. These results highlight the need for discipline-specific injury prevention strategies.

Regarding sports professions, MTSS was the most incident and burden-causing injury in cross-country skiers. In biathlon athletes, ankle sprains were the most common injuries, and they led to the highest injury burden. In Alpine skiers, ACL injuries and MTSS led to the highest-burden, and ankle sprains were detected as the most common occurring injuries. Likewise, ankle sprains were the most common problem for ski jumpers too. Again, ankle sprains were the leading injury in terms of incidence, and knee collateral ligament injuries presented the highest burden, among snowboarders [19]. Although several injury surveillance studies have been conducted in specific snowsport disciplines, epidemiological evidence describing injury incidence and burden across multiple snowsports remains limited, particularly for overuse injuries and sex-specific patterns [20,21].

To sum up, ankle sprains were the highest-incident injuries among women, men, Alpine skiers, biathletes, ski jumpers, and snowboarders, while MTSS was the highest-incident injury among cross-country skiers. The high prevalence of ankle injuries we saw aligns with researchers’ previously reported high prevalence of ankle sprains among snowsport athletes [22]. However, this homogeneity changed for injury burden. Specifically, ACL injuries were the leading burden among women, whereas MTSS injuries were causing the highest number of time-off-weeks among male athletes. By sport type, MTSS injuries had the highest burden for cross-country skiers. However, knee collateral ligament injuries led to the highest burden among snowboarders. Likewise, ACL ruptures represented the highest injury burden among Alpine skiers. Although the majority of our total sample consisted of male cross-country skiers (31 men and 10 women), this finding is consistent with previous evidence showing that female athletes have a higher risk of ACL injury compared with males. Finally, ankle sprains were the highest burden among biathletes and ski jumpers. Knee injuries are severe injuries compared to ankle sprains; this could be the reason for the higher injury burden of knee injuries despite their lower incidences in comparison with ankle sprains [1].

Our study found that the majority of injuries were training injuries rather than competition injuries (Table 2). This aligns with prior injury incidence research in snowsport athletes, which found the incidence of injuries to be higher during training relative to competition (e.g., 73.8 vs. 23.1 in Alpine skiers; 77.8 vs. 22.2 in snowboard slalom; 70.6 vs. 23.5 in snowboard slopestyle; 71.4 vs. 28.6 in ski jumping; and 71.4 vs. 28.9 in biathlon) [23]. One explanation for higher injury incidences during training could be the greater time spent in training compared to competition. The retrospective design of our study might have failed to detect all the competition or training injuries among members of the Turkish National Ski Team because of dynamic team memberships based on who the top performers are. Thus, the study may have failed to gather information about athletes who left the national team for any reason, including but not limited to injury, illness, accident, performance decrements, and end of their athletic career.

The differences in injury patterns by athletes’ sex and by snowsport discipline are indeed significant and have practical implications for injury prevention. For example, ACL ruptures led to the highest injury burden among women in our cohort, a finding that is consistent with reports of higher ACL injury risk in female elite skiers [22]. Women have approximately 1.65 times greater incidence of ACL ruptures than men in World Cup Alpine skiing, and ACL injuries have been noted as a common diagnosis among elite female ski jumpers [22,24,25]. Accordingly, experts recommend that prevention programs specifically target ACL injury reduction in female athletes alongside interventions for other common injuries we identified in women (such as MTSS, hamstring strains, calf strains, Achilles tendon ruptures, anterior tibial pain, and ankle sprains) [26]. In contrast, among male snowsport athletes in our study, overuse injuries like MTSS, as well as ankle sprains and knee collateral ligament injuries, were the most frequent and burdensome injury types. This finding aligns with large-scale epidemiological evidence indicating that ankle and knee sprains and other lower extremity injuries represent the most common injury patterns in predominantly male snowsport populations, accounting for a substantial proportion of all reported injuries [27]. Therefore, injury prevention initiatives should be optimized according to sex-specific injury profiles rather than applying uniform prevention strategies across all athletes. Moreover, each snowsport discipline examined in this study demonstrated a distinct injury profile, underscoring the necessity of tailoring prevention strategies to the specific biomechanical and training demands of each sport. For example, MTSS was most frequently observed among cross-country skiers, ankle sprains predominated in biathletes and ski jumpers, ACL ruptures were most burdensome in Alpine skiers, and knee collateral ligament injuries were particularly prominent among snowboarders. Finally, consistent with previous snowsport injury surveillance studies, the majority of injuries in our cohort occurred during training rather than competition, highlighting training load management and structured preventive interventions during training periods as critical focal points for reducing overall injury incidence.

Similarly, ACL ruptures were the most common diagnosis among women ski jumpers [26]. Therefore, it has been recommended that prevention programs target ACL injury prevention for women athletes [28] in addition to other common and severe injuries identified among elite women athletes in this study, such as MTSS, hamstring strains, calf strains, Achilles ruptures, tibialis anterior pain, and ankle sprains. Among male snowsport athletes, MTSS, ankle sprains, knee collateral ligament injuries, patellar tendinitis, tibialis anterior pain, and hip injuries were the most severe and common injuries. Likewise, ankle and knee sprains and lower leg injuries were found as the highest-incident lower extremity injuries in a large sample (7966 injuries), which was majority men (61%) [27]. Therefore, injury prevention programs should be optimized based on sex-specific injury incidences. It is important to note that researchers across kinesiology disciplines have highlighted that performance and injury susceptibility are influenced by the intertwining of individuals’ sex and gender, which includes their social and cultural context. In the context of sports, sex-related biological factors such as hormonal profiles, joint laxity, and neuromuscular control, together with gender-related aspects such as differences in training opportunities, coaching attention, and role expectations, can lead to different injury patterns and mechanisms between men and women athletes [25]. Therefore, injury prevention programs should account for both biological and sociocultural factors when developing discipline-specific strategies [29]. In the sport domain, the social and cultural context includes sex differences in training opportunities and experiences [30].

Regarding snowsport disciplines, each sport demonstrated distinct injury incidence and burden patterns. MTSS was the most frequent injury among cross-country skiers, ankle sprains among biathletes, ACL ruptures and ankle sprains among Alpine skiers, ankle sprains among ski jumpers, and knee collateral ligament injuries and ankle sprains among snowboarders. These discipline-specific differences highlight the need for tailored prevention strategies focusing on the most common and burdensome injuries within each sport.

MTSS and tibialis anterior pain were frequent among participants in this study. Specifically, tibialis anterior pain can be an early sign of MTSS or anterior compartment syndrome of the lower leg [31]. However, due to the retrospective design of the study, the relationships between tibialis anterior pain, MTSS, and anterior compartment syndrome could not be investigated. Therefore, in addition to adopting program designs to reduce the risk of acute injuries (e.g., ACL ruptures, ankle sprains), injury prevention specialists should also consider injury prevention of anterior compartment syndrome, which is an overuse injury common in snowsports [31,32,33,34,35], when preparing an optimal injury prevention program.

## 5. Limitations

This study has limitations that should be considered when interpreting the findings. First, the retrospective design relies on the completeness of existing medical and federation records and therefore may have missed unreported or minor complaints, despite attempts to confirm injury diagnoses and return-to-sport dates through athlete interviews. In addition, our cohort consisted of athletes registered and available within the national team system during the data-collection period; athletes who left the national team (including because of injury) were not captured, which may have led to underestimation of injury incidence and burden. Second, exposure hours and time-loss duration were estimated from official training/competition schedules and records, and therefore some misclassification remains possible, particularly across disciplines and over long career spans. Third, the sample size was modest overall, and several disciplines included small numbers of athletes, which limits the precision of discipline-specific estimates and generalizability. Finally, contextual and mechanistic information (e.g., weather and snow conditions, equipment factors, training-load progression, and detailed biomechanical or situational circumstances at the time of injury) was not available, precluding analysis of risk factors or causal mechanisms. Consequently, while our study aimed to provide a comprehensive epidemiological overview of lower extremity injuries, the retrospective design and lack of mechanistic data prevented us from reliably distinguishing between acute and overuse injury mechanisms, which should be considered when interpreting the findings related to overuse injuries. Future prospective, multi-center surveillance using standardized exposure tracking and overuse injury monitoring tools could address these limitations.

## 6. Conclusions

Injury incidence and burden patterns differ by sex and snowsport discipline. Ankle sprains were the most frequent injuries overall, whereas MTSS and ACL ruptures accounted for the highest injury burden in terms of time loss. Importantly, ACL injuries were more burdensome among female athletes, MTSS among male athletes, and discipline-specific differences were observed across snowsports. These findings address the study’s aim and contribute to the existing literature by highlighting the importance of considering both injury incidence and burden when designing sex- and sport-specific injury prevention strategies.

## Figures and Tables

**Table 1:** Characteristics of the athletic cohort (mean ± SD and median [IQR]).

Variable	Mean ± SD	Median [IQR]
Age (years)	18.1 ± 2.4	18 [17–20]
Sex, *n* (%)	34 females (34.3%)/65 males (65.7%)	–
Body mass (kg)	59.3 ± 9.5	58 [52–65]
Height (cm)	167 ± 8.5	167 [160–174]
BMI (kg/m^2^)	20.7 ± 2.2	20.4 [19.2–22.1]
Total years of athletic performance (years)	7.3 ± 3.2	7 [5–9]
Age at first injury (years)	15.5 ± 2.3	15 [14–17]
Total membership duration in the national team (years)	2.2 ± 2.8	1 [0–3]
Total training and competition hours during athletic career (hours)	3735.3 ± 2239.5	3400 [2200–4600]

**Table 2:** Injury incidence and burden (mean ± SD [95% CI]) (sorted from highest to lowest overall incidence).

Injuries		Total Injury Incidence per 10,000 h	Total Injury Burden (Number of Time-Off Weeks per 10,000 h)	Competition Injury Incidence per 10,000 h	Training Injury Incidence per 10,000 h
**Ankle Sprains**	**Overall (*n* = 99)**	1.7 ± 4 [0.9, 2.5]	4.4 ± 9.9 [2.5, 6.4]	0.2 ± 1.0 [−0.01, 0.4]	1.6 ± 3.7 [0.9, 2.4]
	*Females* (*n* = 34)	1.3 ± 3 [0.2, 2.3]	2.9 ± 6.7 [0.5, 5.2]	0.2 ± 1.2 [−0.2, 0.6]	1.3 ± 3 [0.2, 2.3]
	*Males* (*n* = 65)	2.0 ± 4.5 [0.9, 3.1]	5.3 ± 11.1 [2.5, 8.0]	0.2 ± 0.9 [−0.04, 0.4]	1.8 ± 4.1 [0.8, 2.8]
	*Cross-country skiers* (*n* = 41)	0.7 ± 2.2 [0.001, 1.4]	2.2 ± 6.6 [0.1, 4.3]	0 ± 0	0.7 ± 2.2 [0.001, 1.4]
	*Biathlon* (*n* = 24)	2.3 ± 5.7 [−0.1, 4.7]	6.9 ± 14.3 [0.8, 12.9]	0.2 ± 1.1 [−0.2, 7.1]	2.1 ± 5.4 [−0.2, 4.4]
	*Alpine skiers* (*n* = 18)	2.8 ± 4.5 [0.5, 5.0]	4.9 ± 7.3 [1.3, 8.5]	0.5 ± 1.7 [−0.3, 1.4]	2.6 ± 4.2 [0.6, 4.7]
	*Ski jumpers* (*n* = 8)	1.6 ± 3.1 [−1.0, 4.2]	6.0 ± 14 [−5.7, 17.8]	0 ± 0	1.6 ± 3.1 [−1, 4.2]
	*Snowboarders* (*n* = 8)	3.2 ± 4.7 [−0.4, 6.9]	5.9 ± 7.5 [−0.3, 12.2]	0.5 ± 1.5 [−0.7, 1.8]	2.7 ± 3.1 [0.1, 5.3]
**MTSS**	**Overall (*n* = 99)**	0.9 ± 3.3 [0.3, 1.6]	9.5 ± 38.7 [1.8, 17.2]	0 ± 0	0.9 ± 3.3 [0.3, 1.6]
	*Females* (*n* = 34)	1.2 ± 4.4 [−0.4, 2.7]	9.1 ± 35.2 [−3.1, 21.4]	0 ± 0	1.2 ± 4.4 [−0.4, 2.7]
	*Males* (*n* = 65)	0.8 ± 2.6 [0.2, 1.4]	9.7 ± 40.7 [−0.4, 19.8]	0 ± 0	0.8 ± 2.6 [0.2, 1.4]
	*Cross-country skiers* (*n* = 41)	1.6 ± 4.7 [0.1, 3.1]	13.4 ± 42.6 [−0.03, 26.9]	0 ± 0	1.6 ± 4.7 [0.1, 3.1]
	*Biathlon* (*n* = 24)	0.4 ± 1.2 [−0.1, 0.9]	2.3 ± 6.5 [−0.4, 5]	0 ± 0	0.4 ± 1.2 [−0.1, 0.9]
	*Alpine skiers* (*n* = 18)	0.8 ± 2.5 [−0.4, 2.1]	16.1 ± 63.6 [−15.5, 47.8]	0 ± 0	0.8 ± 2.5 [−0.4, 2.1]
	*Ski jumpers* (*n* = 8)	0.1 ± 0.4 [−0.2, 0.4]	4.2 ± 11.9 [−5.7, 14.1]	0 ± 0	0.1 ± 0.4 [−0.2, 0.4]
	*Snowboarders* (*n* = 8)	0.2 ± 0.5 [−0.3, 0.6]	1.5 ± 4.3 [−2.1, 5.1]	0 ± 0	0.2 ± 0.5 [−0.3, 0.6]
**Tibialis Anterior Pain**	**Overall (*n* = 99)**	0.8 ± 2.4 [0.4, 1.3]	1.9 ± 6.2 [0.6, 3.1]	0.2 ± 1.2 [−0.1, 0.4]	0.7 ± 2 [0.3, 1.1]
	*Females* (*n* = 34)	0.5 ± 1.7 [−0.1, 1.1]	1.5 ± 5.1 [−0.2, 3.3]	0 ± 0	0.5 ± 1.7 [−0.1, 1.1]
	*Males* (*n* = 65)	1.0 ± 2.7 [0.3, 1.7]	2.0 ± 6.8 [0.4, 3.7]	0.2 ± 1.5 [−0.1, 0.6]	0.8 ± 2.2 [0.3, 1.3]
	*Cross-country skiers* (*n* = 41)	1.3 ± 3 [0.3, 2.2]	3.1 ± 8.6 [0.4, 5.8]	0.3 ± 1.9 [−0.3, 0.9]	1.0 ± 2.4 [0.2, 1.7]
	*Biathlon* (*n* = 24)	1.1 ± 2.9 [−0.1, 2.3]	2.0 ± 5.3 [−0.2, 4.3]	0.1 ± 0.7 [−0.1, 0.4]	1.0 ± 2.4 [−0.004, 2]
	*Alpine skiers* (*n* = 18)	0.2 ± 0.9 [−0.2, 0.7]	0.4 ± 1.8 [−0.5, 1.3]	0 ± 0	0.2 ± 0.9 [−0.2, 0.7]
	*Ski jumpers* (*n* = 8)	0.1 ± 0.4 [−0.2, 0.4]	0.3 ± 0.7 [−0.4, 0.9]	0 ± 0	0.1 ± 0.4 [−0.2, 0.4]
	*Snowboarders* (*n* = 8)	0 ± 0	0 ± 0	0 ± 0	0 ± 0
**Hip/Groin Muscle Injuries**	**Overall (*n* = 99)**	0.8 ± 3.0 [0.2, 1.4]	2.8 ± 10.5 [0.7, 4.8]	0.1 ± 0.5 [−0.1, 0.2]	0.7 ± 2.9 [0.1, 1.3]
	*Females* (*n* = 34)	0.3 ± 1.1 [−0.1, 0.6]	1.1 ± 5 [−0.6, 2.9]	0 ± 0	0.3 ± 1.1 [−0.1, 0.6]
	*Males* (*n* = 65)	1.1 ± 3.6 [0.2, 2]	3.6 ± 12.4 [0.5, 6.7]	0.1 ± 0.6 [−0.1, 0.2]	1.0 ± 3.5 [0.1, 1.9]
	*Cross-country skiers* (*n* = 41)	1.1 ± 3.9 [−0.2, 2.3]	3.7 ± 13.2 [−0.5, 7.9]	0.1 ± 0.8 [−0.1, 0.4]	0.9 ± 3.9 [0.3, 2.2]
	*Biathlon* (*n* = 24)	1.1 ± 3 [−0.2, 2.4]	3.5 ± 11.1 [−1.1, 8.2]	0 ± 0	1.1 ± 3 [−0.2, 2.4]
	*Alpine skiers* (*n* = 18)	0.2 ± 0.8 [−0.2, 0.6]	1.5 ± 6.5 [−1.7, 4.8]	0 ± 0	0.2 ± 0.8 [−0.2, 0.6]
	*Ski jumpers* (*n* = 8)	0 ± 0	0 ± 0	0 ± 0	0 ± 0
	*Snowboarders* (*n* = 8)	0.5 ± 1.5 [−0.7, 1.8]	1.1 ± 3 [−1.5, 3.6]	0 ± 0	0.5 ± 1.5 [−0.7, 1.8]
**Achilles Ruptures**	**Overall (*n* = 99)**	0.5 ± 2.0 [0.1, 0.9]	1.7 ± 6.7 [0.3, 3]	0 ± 0	0.5 ± 2.0 [0.1, 0.9]
	*Females* (*n* = 34)	1.0 ± 3.2 [−0.2, 2.1]	3.0 ± 10.2 [−0.6, 6.5]	0 ± 0	1.0 ± 3.2 [−0.2, 2.1]
	*Males* (*n* = 65)	0.2 ± 0.9 [0.03, 0.5]	1.0 ± 3.8 [0.1, 2]	0 ± 0	0.2 ± 0.9 [0.03, 0.5]
	*Cross-country skiers* (*n* = 41)	0.9 ± 3.0 [−0.1, 1.8]	2.6 ± 9.3 [−0.3, 5.6]	0 ± 0	0.9 ± 3.0 [−0.1, 1.8]
	*Biathlon* (*n* = 24)	0.3 ± 1.2 [−0.2, 0.8]	1.1 ± 4.9 [−1.0, 3.2]	0 ± 0	0.3 ± 1.2 [−0.2, 0.8]
	*Alpine skiers* (*n* = 18)	0.2 ± 0.6 [−0.04, 0.5]	0.9 ± 2.2 [−0.2, 2.0]	0 ± 0	0.2 ± 0.6 [−0.04, 0.5]
	*Ski jumpers* (*n* = 8)	0.1 ± 0.4 [−0.2, 0.4]	2.1 ± 5.9 [−2.9, 7.1]	0 ± 0	0.1 ± 0.4 [−0.2, 0.4]
	*Snowboarders* (*n* = 8)	0 ± 0	0 ± 0	0 ± 0	0 ± 0
**QF Strains**	**Overall (*n* = 99)**	0.4 ± 3.4 [−0.3, 1.1]	0.8 ± 5.2 [−0.3, 1.8]	0.1 ± 1 [−0.1, 0.3]	0.3 ± 2.7 [−0.2, 0.9]
	*Females* (*n* = 34)	0 ± 0	0 ± 0	0 ± 0	0 ± 0
	*Males* (*n* = 65)	0.4 ± 3.4 [−0.4, 1.7]	1.2 ± 6.4 [−0.4, 2.8]	0.2 ± 1.2 [−0.1, 0.5]	0.4 ± 3.3 [−0.4, 1.3]
	*Cross-country skiers* (*n* = 41)	1 ± 5.3 [−0.7, 2.7]	1.7 ± 8 [−0.8, 4.3]	0.3 ± 1.5 [−0.1, 0.8]	0.7 ± 4.2 [−0.7, 2.0]
	*Biathlon* (*n* = 24)	0 ± 0	0 ± 0	0 ± 0	0 ± 0
	*Alpine skiers* (*n* = 18)	0 ± 0	0 ± 0	0 ± 0	0 ± 0
	*Ski jumpers* (*n* = 8)	0 ± 0	0 ± 0	0 ± 0	0 ± 0
	*Snowboarders* (*n* = 8)	0.2 ± 0.6 [−0.3, 0.7]	0.6 ± 1.7 [−0.8, 2.1]	0 ± 0	0.2 ± 0.6 [−0.3, 0.7]
**Patellar Tendinitis**	**Overall (*n* = 99)**	0.4 ± 2.2 [−0.1, 0.8]	3.0 ± 14.9 [0.04, 6]	0 ± 0	0.4 ± 2.2 [−0.1, 0.8]
	*Females (n = 34)*	0.1 ± 0.4 [−0.1, 0.2]	0.8 ± 4.5 [−0.8, 2.3]	0 ± 0	0.1 ± 0.4 [−0.1, 0.2]
	*Males* (*n* = 65)	0.6 ± 2.7 [−0.1, 1.2]	4.2 ± 18 [−0.3, 8.7]	0 ± 0	0.6 ± 2.7 [−0.1, 1.2]
	*Cross-country skiers* (*n* = 41)	0.1 ± 0.8 [−0.1, 0.4]	1.0 ± 6.5 [−1.0, 3.1]	0 ± 0	0.1 ± 0.8 [−0.1, 0.4]
	*Biathlon* (*n* = 24)	0.2 ± 0.8 [−0.1, 0.6]	3.4 ± 12.5 [−1.8, 8.7]	0 ± 0	0.2 ± 0.8 [−0.1, 0.6]
	*Alpine skiers* (*n* = 18)	1.4 ± 4.9 [−1.1, 3.8]	7.4 ± 29.4 [−7.3, 22]	0 ± 0	1.4 ± 4.9 [−1.1, 3.8]
	*Ski jumpers* (*n* = 8)	0.4 ± 0.7 [−0.2, 1.0]	5.1 ± 12.6 [−5.4, 15.7]	0 ± 0	0.4 ± 0.7 [−0.2, 1.0]
	*Snowboarders* (*n* = 8)	0 ± 0	0 ± 0	0 ± 0	0 ± 0
**Knee Collateral Ligaments Injuries**	**Overall (*n* = 99)**	0.4 ± 1.3 [0.1, 0.6]	3.8 ± 23.4 [−0.9, 8.5]	0.1 ± 0.6 [−0.1, 0.2]	0.4 ± 1.3 [0.1, 0.6]
	*Females* (*n* = 34)	0.3 ± 1.5 [−0.2, 0.8]	2.4 ± 11.6 [−1.7, 6.4]	0 ± 0	0.3 ± 1.5 [−0.2, 0.8]
	*Males* (*n* = 65)	0.4 ± 1.2 [0.1, 0.7]	4.5 ± 27.7 [−2.3, 11.4]	0.1 ± 0.7 [−0.1, 0.3]	0.4 ± 1.2 [0.1, 0.7]
	*Cross-country skiers* (*n* = 41)	0.1 ± 0.7 [−0.1, 0.3]	0.2 ± 1.5 [−0.2, 0.7]	0 ± 0	0.1 ± 0.7 [−0.1, 0.3]
	*Biathlon* (*n* = 24)	0.5 ± 1.5 [−0.1, 1.2]	2.4 ± 6.5 [−0.4, 5.1]	0.2 ± 1.1 [−0.2, 0.7]	0.5 ± 1.5 [−0.1, 1.2]
	*Alpine skiers* (*n* = 18)	0.8 ± 2.1 [−0.3, 1.8]	4.8 ± 15.8 [−3.1, 12.7]	0 ± 0	0.8 ± 2.1 [−0.3, 1.8]
	*Ski jumpers* (*n* = 8)	0 ± 0	0 ± 0	0 ± 0	0 ± 0
	*Snowboarders* (*n* = 8)	0.5 ± 1.5 [−0.7, 1.8]	27.8 ± 78.6 [−37.9, 93.5]	0 ± 0	0.5 ± 1.5 [−0.7, 1.8]
**Hamstring Strains**	**Overall (*n* = 99)**	0.3 ± 1.2 [0.1, 0.6]	2.8 ± 16 [−0.4, 6.0]	0 ± 0	0.3 ± 1.2 [0.1, 0.6]
	*Females* (*n* = 34)	0.8 ± 1.9 [0.1, 1.4]	6.4 ± 25 [−2.4, 15.1]	0 ± 0	0.8 ± 1.9 [0.1, 1.4]
	*Males* (*n* = 65)	0.1 ± 0.7 [−0.1, 0.3]	1.0 ± 7.8 [−0.9, 2.9]	0 ± 0	0.1 ± 0.7 [−0.1, 0.3]
	*Cross-country skiers* (*n* = 41)	0.4 ± 1.3 [−0.1, 0.8]	5.5 ± 24.4 [−2.2, 13.2]	0 ± 0	0.4 ± 1.3 [−0.1, 0.8]
	*Biathlon* (*n* = 24)	0.3 ± 1.6 [−0.3, 1.0]	0.7 ± 3.2 [−0.7, 2.0]	0 ± 0	0.3 ± 1.6 [−0.3, 1.0]
	*Alpine skiers* (*n* = 18)	0.5 ± 1.1 [−0.01, 1.1]	2.3 ± 5.3 [−0.3, 4.9]	0 ± 0	0.5 ± 1.1 [−0.01, 1.1]
	*Ski jumpers* (*n* = 8)	0 ± 0	0 ± 0	0 ± 0	0 ± 0
	*Snowboarders* (*n* = 8)	0 ± 0	0 ± 0	0 ± 0	0 ± 0
**Meniscus Tears**	**Overall (*n* = 99)**	0.3 ± 1.3 [0.1, 0.6]	2.5 ± 11.7 [0.1, 4.8]	0 ± 0.0001 [0, 0.0001]	0.3 ± 1.2 [0.1, 0.6]
	*Females* (*n* = 34)	0.2 ± 1.2 [−0.2, 0.6]	2.7 ± 15.5 [−2.7, 8.1]	0 ± 0	0.2 ± 1.2 [−0.2, 0.6]
	*Males* (*n* = 65)	0.4 ± 1.3 [0.03, 0.7]	2.4 ± 9.3 [0.1, 4.7]	0 ± 0.0001 [0, 0.0001]	0.4 ± 1.3 [0.03, 0.7]
	*Cross-country skiers* (*n* = 41)	0.5 ± 1.6 [−0.03, 1.0]	4.5 ± 17.1 [−1.0, 9.9]	0 ± 0	0.5 ± 1.6 [−0.03, 1.0]
	*Biathlon* (*n* = 24)	0.3 ± 1.2 [−0.3, 0.8]	1 ± 4.9 [−1.1, 3.1]	0 ± 0.0002	0.3 ± 1.2 [−0.3, 0.8]
	*Alpine skiers* (*n* = 18)	0.2 ± 0.7 [−0.2, 0.5]	0.6 ± 2.6 [−0.7, 1.9]	0 ± 0	0.2 ± 0.7 [−0.2, 0.5]
	*Ski jumpers* (*n* = 8)	0.1 ± 0.4 [−0.2, 0.4]	3.4 ± 9.7 [−4.7, 11.5]	0 ± 0	0.1 ± 0.4 [−0.2, 0.4]
	*Snowboarders* (*n* = 8)	0 ± 0	0 ± 0	0 ± 0	0 ± 0
**ACL Ruptures**	**Overall (*n* = 99)**	0.2 ± 1.2 [0.003, 0.5]	7.0 ± 39.3 [−0.8, 14.9]	0.01 ± 0.1 [−0.01, 0.03]	0.2 ± 1.2 [−0.003, 0.5]
	*Females* (*n* = 34)	0.4 ± 1.3 [−0.1, 0.8]	16.2 ± 64.5 [−6.32, 38.7]	0 ± 0	0.4 ± 1.3 [−0.1, 0.8]
	*Males* (*n* = 65)	0.2 ± 1.1 [−0.1, 0.4]	2.3 ± 12.2 [−0.8, 5.3]	0.01 ± 0.1 [−0.01, 0.04]	0.2 ± 1.1 [−0.1, 0.4]
	*Cross-country skiers* (*n* = 41)	0 ± 0	0 ± 0	0 ± 0	0 ± 0
	*Biathlon* (*n* = 24)	0.04 ± 0.2 [−0.04, 0.1]	0.3 ± 1.5 [−0.3, 0.9]	0.04 ± 0.2 [−0.04, 0.1]	0 ± 0
	*Alpine skiers* (*n* = 18)	1.2 ± 2.5 [−0.1, 2.4]	34.3 ± 87.2 [−9.1, 77.6]	0 ± 0	1.2 ± 2.5 [−0.1, 2.4]
	*Ski jumpers* (*n* = 8)	0 ± 0	0 ± 0	0 ± 0	0 ± 0
	*Snowboarders* (*n* = 8)	0.2 ± 0.6 [−0.3, 0.7]	9.2 ± 26 [−12.5, 30.9]	0 ± 0	0.2 ± 0.6 [−0.3, 0.7]
**PFPS**	**Overall (*n* = 99)**	0.2 ± 1.0 [−0.02, 0.4]	1.0 ± 7.4 [−0.5, 2.4]	0 ± 0	0.2 ± 1.0 [−0.02, 0.4]
	*Females* (*n* = 34)	0 ± 0	0 ± 0	0 ± 0	0 ± 0
	*Males* (*n* = 65)	0.3 ± 1.2 [−0.04, 0.5]	1.5 ± 9.1 [−0.8, 3.7]	0 ± 0	0.3 ± 1.2 [−0.04, 0.5]
	*Cross-country skiers* (*n* = 41)	0.4 ± 1.5 [−0.1, 0.9]	2.3 ± 11.4 [−1.3, 5.9]	0 ± 0	0.4 ± 1.5 [−0.1, 0.9]
	*Biathlon* (*n* = 24)	0 ± 0	0 ± 0	0 ± 0	0 ± 0
	*Alpine skiers* (*n* = 18)	0 ± 0	0 ± 0	0 ± 0	0 ± 0
	*Ski jumpers* (*n* = 8)	0 ± 0	0 ± 0	0 ± 0	0 ± 0
	*Snowboarders* (*n* = 8)	0 ± 0	0 ± 0	0 ± 0	0 ± 0
**Calf Strains**	**Overall (*n* = 99)**	0.2 ± 0.9 [−0.01, 0.3]	1.1 ± 7.7 [−0.4, 2.7]	0 ± 0	0.2 ± 0.9 [−0.01, 0.3]
	*Females* (*n* = 34)	0.5 ± 1.4 [−0.02, 1.0]	3.3 ± 13 [−1.2, 7.8]	0 ± 0	0.5 ± 1.4 [−0.02, 1.0]
	*Males* (*n* = 65)	0 ± 0	0 ± 0	0 ± 0	0 ± 0
	*Cross-country skiers* (*n* = 41)	0.4 ± 1.3 [−0.01, 0.8]	2.7 ± 11.8 [−1.0, 6.5]	0 ± 0	0.4 ± 1.3 [−0.01, 0.8]
	*Biathlon* (*n* = 24)	0 ± 0	0 ± 0	0 ± 0	0 ± 0
	*Alpine skiers* (*n* = 18)	0 ± 0	0 ± 0	0 ± 0	0 ± 0
	*Ski jumpers* (*n* = 8)	0 ± 0	0 ± 0	0 ± 0	0 ± 0
	*Snowboarders* (*n* = 8)	0 ± 0	0 ± 0	0 ± 0	0 ± 0

Abbreviations: ACL: Anterior cruciate ligament; CI: confidence interval; MTSS: medial tibial stress syndrome; PFPS: patellofemoral pain syndrome; QF: quadriceps femoris.

**Table 3:** Top five injuries based on incidence and burden in females, males, cross-country, biathlon and Alpine skiers, ski jumpers, snowboarders, and total sample.

	Incidence (Injuries per Athlete per 10,000 h)		Burden (Time-Off Weeks per Athletes per 10,000 h)	
**Total Sample**	Ankle Sprains	1.7 ± 4.0 [0.9, 2.5]	MTSS	9.5 ± 38.7 [1.8, 17.2]
	MTSS	0.9 ± 3.3 [0.3, 1.6]	ACL Ruptures	7.0 ± 39.3 [−0.8, 14.9]
	Tibialis Anterior Pain	0.8 ± 2.4 [0.4, 1.3]	Ankle Sprains	4.4 ± 9.9 [2.5, 6.4]
	Hip Injuries	0.8 ± 3 [0.2, 1.4]	Knee Collateral Ligament Injuries	3.8 ± 23.4 [−0.9, 8.5]
	Achilles Ruptures	0.5 ± 2 [0.1, 0.9]	Patellar Tendinitis	3.0 ± 14.9 [0.04, 6.0]
**Females**	Ankle Sprains	1.3 ± 3 [0.2, 2.3]	ACL Ruptures	16.2 ± 64.5 [−6.32, 38.7]
	MTSS	1.2 ± 4.4 [−0.4, 2.7]	MTSS	9.1 ± 35.2 [−3.1, 21.4]
	Achilles Ruptures	1.0 ± 3.2 [−0.2, 2.1]	Hamstring Strains	6.4 ± 25 [−2.4, 15.1]
	Hamstring Strains	0.8 ± 1.9 [0.1, 1.4]	Calf Strains	3.3 ± 13 [−1.2, 7.8]
	Tibialis Anterior Pain	0.5 ± 1.7 [−0.1, 1.1]	Achilles Ruptures	3.0 ± 10.2 [−0.6, 6.5]
	Calf Strains	0.5 ± 1.4 [−0.02, 1.0]		
**Males**	Ankle Sprains	2.0 ± 4.5 [0.9, 3.1]	MTSS	9.7 ± 40.7 [−0.4, 19.8]
	Hip Injuries	1.1 ± 3.6 [0.2, 2.0]	Ankle Sprains	5.3 ± 11.1 [2.5, 8.0]
	Tibialis Anterior Pain	1.0 ± 2.7 [0.3, 1.7]	Knee Collateral Ligament Injuries	4.5 ± 27.7 [−2.3, 11.4]
	MTSS	0.8 ± 2.6 [0.2, 1.4]	Patellar Tendinitis	4.2 ± 18 [−0.3, 8.7]
	Patellar Tendinitis	0.6 ± 2.7 [−0.1, 1.2]	Tibialis Anterior Pain	2 ± 6.8 [0.4, 3.7]
**Cross-country skiers**	MTSS	1.6 ± 4.7 [0.1, 3.1]	MTSS	13.4 ± 42.6 [−0.03, 26.9]
	Tibialis Anterior Pain	1.3 ± 3 [0.3, 2.2]	Hamstring Strains	5.5 ± 24.4 [−2.2, 13.2]
	Hip Injuries	1.1 ± 3.9 [−0.2, 2.3]	Meniscus Tears	4.5 ± 17.1 [−1.0, 9.9]
	QF Strains	1.0 ± 5.3 [−0.7, 2.7]	Hip Injuries	3.7 ± 13.2 [−0.5, 7.9]
	Achilles Rupture	0.9 ± 3 [−0.1, 1.8]	Tibialis Anterior Pain	3.1 ± 8.6 [0.4, 5.8]
**Biathlon**	Ankle Sprains	2.3 ± 5.7 [−0.1, 4.7]	Ankle Sprains	6.9 ± 14.3 [0.8, 12.9]
	Tibialis Anterior Pain	1.1 ± 2.9 [−0.1, 2.3]	Hip Injuries	3.5 ± 11.1 [−1.1, 8.2]
	Hip Injuries	1.1 ± 3 [−0.2, 2.4]	Patellar Tendinitis	3.4 ± 12.5 [−1.8, 8.7]
	Knee Collateral Ligament Ruptures	0.5 ± 1.5 [−0.1, 1.2]	Knee Collateral Ligament Injuries	2.4 ± 6.5 [−0.4, 5.1]
	MTSS	0.4 ± 1.2 [−0.1, 0.9]	MTSS	2.3 ± 6.5 [−0.4, 5.0]
**Alpine skiers**	Ankle Sprains	2.8 ± 4.5 [0.5, 5.0]	ACL Ruptures	34.3 ± 87.2 [−9.1, 77.6]
	Patellar Tendinitis	1.4 ± 4.9 [−1.1, 3.8]	MTSS	16.1 ± 63.6 [−15.5, 47.8]
	ACL Ruptures	1.2 ± 2.5 [−0.1, 2.4]	Patellar Tendinitis	7.4 ± 29.4 [−7.3, 22.0]
	Knee Collateral Ligament Ruptures	0.8 ± 2.1 [−0.3, 1.8]	Ankle Sprains	4.9 ± 7.3 [1.3, 8.5]
	MTSS	0.8 ± 2.5 [−0.4, 2.1]	Knee Collateral Ligament Injuries	4.8 ± 15.8 [−3.1, 12.7]
**Ski jumpers**	Ankle Sprains	1.6 ± 3.1 [−1.0, 4.2]	Ankle Sprains	6.0 ± 14 [−5.7, 17.8]
	Patellar Tendinitis	0.4 ± 0.7 [−0.2, 1.0]	Patellar Tendinitis	5.1 ± 12.6 [−5.4, 15.7]
	MTSS	0.1 ± 0.4 [−0.2, 0.4]	MTSS	4.2 ± 11.9 [−5.7, 14.1]
	Tibialis Anterior Pain	0.1 ± 0.4 [−0.2, 0.4]	Meniscus Tears	3.4 ± 9.7 [−4.7, 11.5]
	Achilles Ruptures	0.1 ± 0.4 [−0.2, 0.4]	Achilles Ruptures	2.1 ± 5.9 [−2.9, 7.1]
**Snowboarders**	Ankle Sprains	3.2 ± 4.7 [−0.4, 6.9]	Knee Collateral Ligament Injuries	27.8 ± 78.6 [−37.9, 93.5]
	Hip Injuries	0.5 ± 1.5 [−0.7, 1.8]	ACL Ruptures	9.2 ± 26 [−12.5, 30.9]
	Knee Collateral Ligament Injuries	0.5 ± 1.5 [−0.7, 1.8]	Ankle Sprains	5.9 ± 7.5 [−0.3, 12.2]
	ACL Ruptures	0.2 ± 0.6 [−0.3, 0.7]	MTSS	1.5 ± 4.3 [−2.1, 5.1]
	QF Strains	0.2 ± 0.6 [−0.3, 0.7]	Hip Injuries	1.1 ± 3 [−1.5, 3.6]
	MTSS	0.2 ± 0.5 [−0.3, 0.6]		

Abbreviations: ACL: Anterior cruciate ligament; CI: confidence interval; MTSS: medial tibial stress syndrome; PFPS: patellofemoral pain syndrome; QF: quadriceps femoris.

## Data Availability

The original contributions presented in this study are included in the article. Further inquiries can be directed to the corresponding author.

## References

[1] Xu Y., Yang C., Yang Y., Zhang X., Zhang S., Zhang M., Liu L., Fu W. (2020). A Narrative Review of Injury Incidence, Location, and Injury Factor of Elite Athletes in Snowsport Events. Front. Physiol..

[2] Shea K.G., Archibald-Seiffer N., Murdock E., Grimm N.L., Jacobs J.C., Willick S., Van Houten H. (2014). Knee Injuries in Downhill Skiers: A 6-Year Survey Study. Orthop. J. Sports Med..

[3] Worth S.G.A., Reid D.A., Howard A.B., Henry S.M. (2019). Injury Incidence in Competitive Cross-Country Skiers: A Prospective Cohort Study. Int. J. Sports Phys. Ther..

[4] Hisdal E., Bahr R. (2019). FIS Injury Surveillance System 2006–2019.

[5] Wu Y., Dai R., Yan W., Ren S., Ao Y. (2023). Characteristics of Sports Injuries in Athletes During the Winter Olympics: A Systematic Review and Meta-analysis. Orthop. J. Sports Med..

[6] Clarsen B., Myklebust G., Bahr R. (2013). Development and validation of a new method for the registration of overuse injuries in sports injury epidemiology: The Oslo Sports Trauma Research Centre (OSTRC) overuse injury questionnaire. Br. J. Sports Med..

[7] Farelli A.D. (2011). Sport Participation: Health Benefits, Injuries and Psychological Effects.

[8] Finch C.F. (1997). An overview of some definitional issues for sports injury surveillance. Sports Med..

[9] Franco M.F., Madaleno F.O., de Paula T.M.N., Ferreira T.V., Pinto R.Z., Resende R.A. (2021). Prevalence of overuse injuries in athletes from individual and team sports: A systematic review with meta-analysis and GRADE recommendations. Braz. J. Phys. Ther..

[10] von Rosen P., Heijne A. (2018). Substantial injuries influence ranking position in young elite athletes of athletics, cross-country skiing and orienteering. Scand. J. Med. Sci. Sports.

[11] von Rosen P., Heijne A., Frohm A., Fridén C., Kottorp A. (2018). High Injury Burden in Elite Adolescent Athletes: A 52-Week Prospective Study. J. Athl. Train..

[12] Cook J., Finch C.F. (2011). The long-term impact of overuse injuries on life-long participation in sport and health status. Sport Participation-Health Benefits, Injuries and Pyschological Effects.

[13] Bahr R. (2009). No injuries, but plenty of pain? On the methodology for recording overuse symptoms in sports. Br. J. Sports Med..

[14] Hildebrandt C., Oberhoffer R., Raschner C., Müller E., Fink C., Steidl-Müller L. (2021). Training load characteristics and injury and illness risk identification in elite youth ski racing: A prospective study. J. Sport. Health Sci..

[15] van Mechelen W. (1997). The severity of sports injuries. Sports Med..

[16] Palmer-Green D., Fuller C., Jaques R., Hunter G. (2013). The Injury/Illness Performance Project (IIPP): A novel epidemiological approach for recording the consequences of sports injuries and illnesses. J. Sports Med..

[17] Ekstrand J., Bengtsson H., Walden M., Davison M., Hagglund M. (2022). Still poorly adopted in male professional football: But teams that used the Nordic Hamstring Exercise in team training had fewer hamstring injuries—A retrospective survey of 17 teams of the UEFA Elite Club Injury Study during the 2020–2021 season. BMJ Open Sport Exerc. Med..

[18] Fu X.-L., Du L., Song Y.-P., Chen H.-L., Shen W.-Q. (2022). Incidence of injuries in professional snow sports: A systematic review and meta-analysis. J. Sport Health Sci..

[19] Kim S., Endres N.K., Johnson R.J., Ettlinger C.F., Shealy J.E. (2012). Snowboarding injuries: Trends over time and comparisons with alpine skiing injuries. Am. J. Sports Med..

[20] Injury I.O.C., Group I.E.C., Bahr R., Clarsen B., Derman W., Dvorak J., Emery C.A., Finch C.F., Hägglund M., Junge A. (2020). International Olympic Committee consensus statement: Methods for recording and reporting of epidemiological data on injury and illness in sports 2020 (including the STROBE extension for sports injury and illness surveillance (STROBE-SIIS)). Orthop. J. Sports Med..

[21] Andersson S.H., Bahr R., Clarsen B., Myklebust G. (2017). Preventing overuse shoulder injuries among throwing athletes: A cluster-randomised controlled trial in 660 elite handball players. Br. J. Sports Med..

[22] Rotllan C., Viscor G. (2023). Winter Sports Injuries in Elite Female Athletes: A Narrative Review. Int. J. Environ. Res. Public Health.

[23] Soligard T., Steffen K., Palmer-Green D., Aubry M., Grant M.E., Meeuwisse W., Mountjoy M., Budgett R., Engebretsen L. (2015). Sports injuries and illnesses in the Sochi 2014 Olympic Winter Games. Br. J. Sports Med..

[24] Barth M., Platzer H., Giger A., Nachbauer W., Schröcksnadel P. (2020). Acute on-snow severe injury events in elite alpine ski racing from 1997 to 2019: The Injury Surveillance System of the Austrian Ski Federation. Br. J. Sports Med..

[25] Zhang Y., Hu Z., Li B., Qiu X., Li M., Meng X., Kim S., Kim Y. (2023). Gender differences in lower extremity stiffness during a single-leg landing motion in badminton. Bioengineering.

[26] Stenseth O.M.R., Barli S.F., Martin R.K., Engebretsen L. (2022). Injuries in elite women’s ski jumping: A cohort study following three International Ski Federation (FIS) World Cup seasons from 2017–2018 to 2019–2020. Br. J. Sports Med..

[27] Ueland O., Kopjar B. (1998). Occurrence and trends in ski injuries in Norway. Br. J. Sports Med..

[28] Palmer-Green D., Elliott N. (2015). Sports injury and illness epidemiology: Great Britain Olympic Team (TeamGB) surveillance during the Sochi 2014 Winter Olympic Games. Br. J. Sports Med..

[29] Lewthwaite R., Wulf G. (2010). Grand challenge for movement science and sport psychology: Embracing the social-cognitive-affective-motor nature of motor behavior. Front. Psychol..

[30] Finestone A.S., Milgrom C., Yanovich R., Evans R., Constantini N., Moran D.S. (2014). Evaluation of the performance of females as light infantry soldiers. BioMed. Res. Int..

[31] Federolf P., Bakker E. (2012). Muscle activation characteristics in cross-country skiers with a history of anterior compartment pain. Sports Biomech..

[32] Clanton T.O., Solcher B.W. (1994). Chronic leg pain in the athlete. Clin. Sports Med..

[33] Gertsch P., Borgeat A., Wälli T. (1987). New cross-country skiing technique and compartment syndrome. Am. J. Sports Med..

[34] Lawson S.K., Reid D.C., Wiley J.P. (1992). Anterior compartment pressures in cross-country skiers. A comparison of classic and skating skis. Am. J. Sports Med..

[35] Styf J. (1988). Diagnosis of exercise-induced pain in the anterior aspect of the lower leg. Am. J. Sports Med..

